# Five Key Papers About Emergency Department Fall Evaluation: A Curated Collection for Emergency Physicians

**DOI:** 10.7759/cureus.17717

**Published:** 2021-09-04

**Authors:** Sung-Ho Kim, Masaya Higuchi, Yuichiro Ishigami, Go Makishi, Masafumi Tada, Seikei Hibino, Michael Gottlieb, Sangil Lee

**Affiliations:** 1 Emergency Medicine, Rinku General Hospital, Osaka, JPN; 2 Trauma and Critical Care, Senshu Trauma and Critical Care Center, Osaka, JPN; 3 Palliative Care and Geriatric Medicine, Massachusetts General Hospital, Boston, USA; 4 Transitional and Palliative Care, Aso Iizuka Hospital, Fukuoka, JPN; 5 Emergency Medicine, Seirei Mikatahara General Hospital, Shizuoka, JPN; 6 Health Promotion and Human Behavior, Kyoto University Graduate School of Medicine/School of Public Health, Kyoto, JPN; 7 Neurology-Emergency Medicine, Nagoya City University East Medical Center, Nagoya, JPN; 8 Emergency Medicine, University of Minnesota, Minneapolis, USA; 9 Emergency Medicine, Rush University Medical Center, Chicago, USA; 10 Emergency Medicine, University of Iowa Carver College of Medicine, Iowa, USA

**Keywords:** curated collection, fall, emergency department, evaluation, older adults, geriatric, education, modified delphi method

## Abstract

The evaluation of patients who have experienced a fall has been an integral part of geriatric emergency care. All physicians who engage in the care of the geriatric population in acute settings need to familiarize themselves with the current literature on this topic. However, it can be challenging to navigate the large body of literature on this topic. The purpose of this article is to identify and summarize the key studies that can be helpful for faculty interested in an evidence-based fall evaluation.

The authors compiled a list of key papers on emergency department (ED) based upon a structured literature search supplemented with suggestions by key informants and an open call on social media; 32 studies on ED evaluation were identified. Our authorship group then engaged in a modified Delphi technique to develop consensus on the most important studies about fall evaluation for emergency physicians. This process eventually resulted in the selection of the top five articles on fall evaluation. Additionally, we summarize these studies with regard to their relevance to emergency medicine (EM) trainees and junior faculty.

Evaluation of older patients with a history of falls is a challenging but crucial component of EM training. We believe our review will be educational for junior and senior EM faculty to better understand these patients' care and to design an evidence-based practice.

## Introduction and background

Falls are a common reason for presentation to the emergency department (ED). Fall injuries and related healthcare use among older adults are increasing in the United States [1]. Each year, three million older people are treated in EDs for injuries related to falls [2]. In 2015, the total medical cost for falls amounted to more than $50 billion [3]. Moreover, falls are associated with high mortality rates [4].

However, there are several causes of falls: syncope, polypharmacy, impaired gait, adverse effects of medications, immobility, and physical inactivity. A fall can be seen as a manifestation of frailty, and it is common to find several underlying medical conditions in fall-related ED visits. Thus, emergency physicians must engage in comprehensive evaluations when examining an older adult with a fall. Emergency physicians also need to understand how to perform an adequate risk assessment for falls among older adults to prevent future falls. Those evaluations are challenging for both resident and attending physicians, and high-quality training in falls, geriatrics, and evidence-based medicine are needed. In light of this, this article seeks to summarize the top five key articles in the literature on falls and discuss their applicability for emergency physicians.

## Review

Methods

The use of the existing literature for resident and student education is a key component of the evidence-based medicine curriculum. We used a modified Delphi method, which has been previously reported, to evaluate the top five articles suggested for inclusion [5-8].

This study was conducted between June 2019 and November 2020. Reviewers were invited via a mailing list of nonprofit organizations promoting emergency medical care in Japan [9]. Reviewers consisted of three senior and four junior emergency medicine (EM) physicians, palliative care physicians, and geriatricians. Senior faculty were defined as more than 10 years post-graduation, while junior faculty were defined as less than 10 years. The online meeting on the video platform Zoom^TM^ (Zoom Video Communications Inc., San Jose, CA) involved junior faculty in EM and mentors who are senior-level faculty physicians in EM and geriatrics. To collect articles, we searched PubMed for the following Medical Subject Headings (MeSH) terms: "emergency medicine", "accidental falls", "falls", "evaluation", and "assessment." The authors also reached out to experts and identified key literature. This was further augmented with a call for articles on Twitter by using the hashtags #fall and #ED. After combining the above articles, we reviewed the abstracts to identify articles focused on fall patients in the ED. Articles not related to the ED or falls were excluded.

After the initial articles were identified, we engaged in three rounds of voting. During the first round of voting, participants read each article and scored it on a 1-7 Likert scale, with 1 representing "not relevant at all" and 7 representing "very relevant." Votes from round one were subsequently compiled, and the distribution of scores was shared with participants in round two. During round two, participants were asked if each article should be included. While the end goal was to identify the top five articles, participants were allowed to choose more than five articles in this round. Votes from the second round were compiled, and the percentage of participants who thought each article should be included was shared with participants for the third round. During the third round, participants were asked to choose only the five articles that they thought were most relevant to be included in the manuscript. As there were more than five articles, we repeated the same selection process during the fourth round. Five articles were chosen as an a priori threshold by group consensus as a reasonable number to introduce the residents to the topic without overwhelming them.

Results

**Figure 1:**
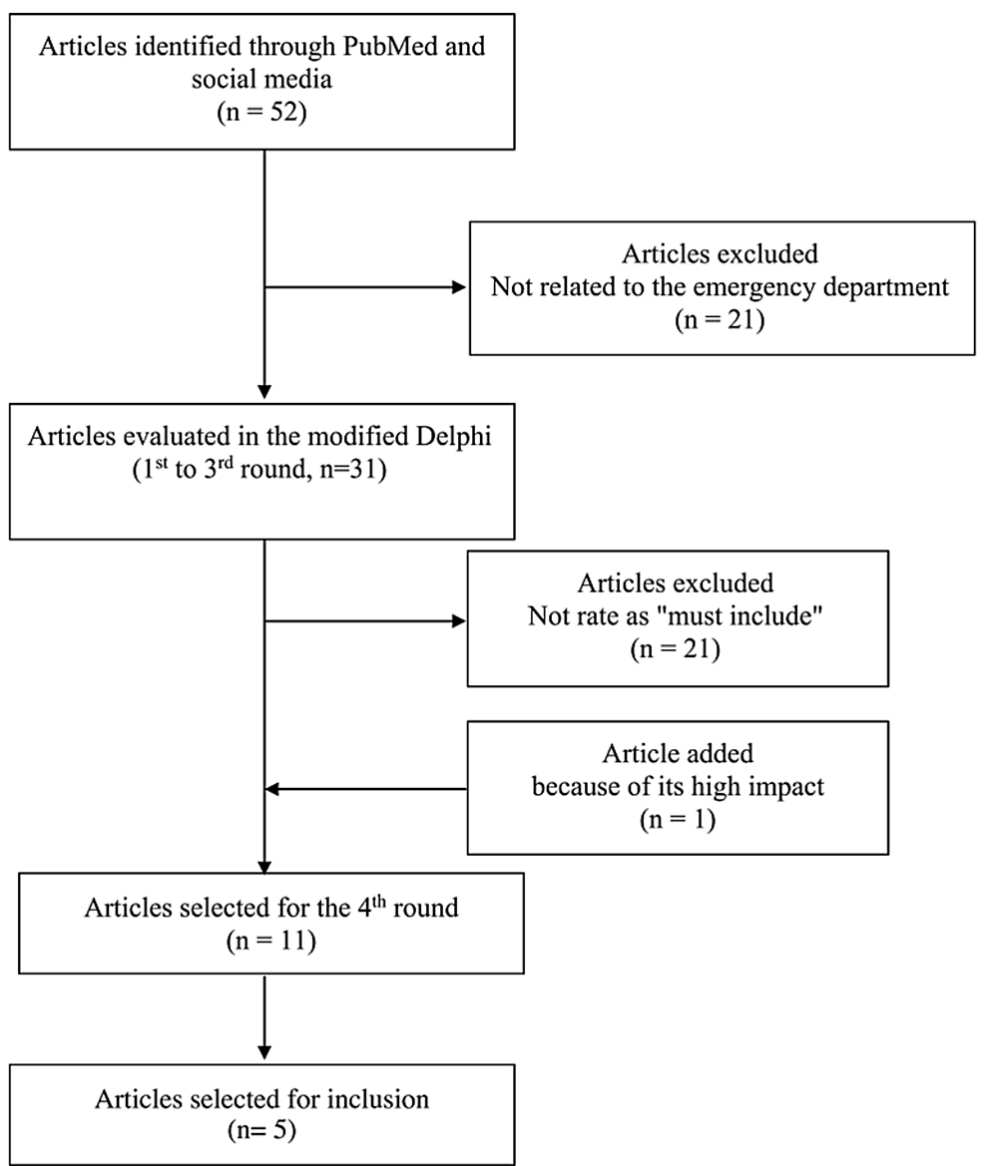
Article selection process

Figure 1 shows the flow chart of article selection in our review. Of 52 identified articles, 21 were excluded as they were not related to the ED.

We identified a total of 32 articles, which were narrowed down to five key papers using the modified Delphi methodology [10-41]. Table 1 shows all identified articles with ratings.

**Table 1:** Reviewer ratings for all 32 articles SD: standard deviation

Citation	Round 1 initial mean scores (SD); max score: 7	Round 2: percentage by raters	Round 3: percentage by raters	Round 4: percentage by raters	Top five articles
Tirrell et al. [10]	5.6 (0.5)	100	100	100	1st
Bhangu et al. [11]	5.4 (0.5)	85.7	85.7	85.7	2nd (tie)
Carpenter et al. [12]	4.7 (1.8)	71.4	85.7	85.7	2nd (tie)
Jeanmonod et al. [13]	5.4 (1.3)	85.7	85.7	85.7	2nd (tie)
Goldberg et al. [14]	-	-	-	85.7	2nd (tie)
Banerjee et al. [15]	4.4 (1.4)	42.9	0	NA	
Baraff et al. [16]	4.0 (1.2)	0	0	NA	
Baraff LJ, et al. [17]	4.3 (1.1)	28.6	0	NA	
Carpenter et al. [18]	5.0 (2.2)	57.1	42.9	20	
Carpenter et al. [19]	5.0 (1.0)	28.6	0	NA	
Caterino et al. [20]	3.6 (1.3)	14.3	0	NA	
Eagles et al. [21]	4.3 (0.8)	14.3	0	NA	
Goldberg et al. [22]	3.4 (1.1)	0	0	NA	
Harper et al. [23]	3.9 (1.5)	0	0	NA	
Harper et al. [24]	3.9 (1.5)	0	0	NA	
Huded et al. [25]	4.6 (1.4)	50	0	NA	
Hwang et al. [26]	4.4 (1.5)	33.3	14.3	20	
Jawa et al. [27]	5.1 (0.9)	42.9	0	NA	
Kara et al. [28]	4.3 (1.5)	0	0	NA	
Kim et al. [29]	4.9 (0.9)	28.6	0	NA	
Labib et al. [30]	5.1 (1.1)	57.1	42.9	20	
McMahon et al. [31]	4.9 (1.6)	14.3	0	NA	
Miró et al. [32]	4.9 (1.1)	28.6	14.3	20	
Patterson et al. [33]	3.7 (0.5)	0	0	NA	
Pfortmueller et al. [34]	4.9 (0.9)	0	0	NA	
Polinder et al. [35]	4.3 (1.1)	0	0	NA	
Schoenenberger et al. [36]	4.6 (1.3)	28.6	14.3	0	
Schrijver et al. [37]	4.4 (0.8)	0	0	NA	
Southerland et al. [38]	4.6 (1.3)	28.6	14.3	20	
Tan et al. [39]	4.0 (0.8)	0	0	NA	
Terrell et al. [40]	3.4 (1.0)	0	0	NA	
Yu et al. [41]	4.6 (0.5)	28.6	0	NA	

Discussion

Based on our review and modified Delphi process, we identified five articles of interest and relevance to junior-level EM providers. Further, we added commentary below to explain the relevance of these studies to trainees and highlight teaching points for senior EM faculty who teach these concepts to trainees. Here, we provide a summary of the articles:

1. Tirrell G, Sri-on J, Lipsitz LA, Camargo CA, Jr., Kabrhel C, Liu SW: Evaluation of Older Adult Patients With Falls in The Emergency Department: Discordance With National Guidelines. Acad Emerg Med. 2015, 22:461-467 [10]

Summary: This study evaluated how the current clinical evaluation of older adults presenting to the ED with a fall is concordant with the geriatric ED guidelines. This study was conducted at an urban level-one trauma center teaching hospital ED. The data were collected retrospectively by chart review. Patients who were 65 years and older who presented to the ED with falls were included. The authors selected the items to evaluate from the national guidelines. The mean age of patients was 80 years. Patients receiving a greater proportion of the recommended guideline assessments were more likely to be older, have higher Charlson Comorbidity Index scores, and reside in assisted living than those patients who had less comprehensive evaluations [10]. The cause and location of falls were the most frequently reported items in ED documentation at 85% and 81%, respectively [10]. An evaluation of gait, balance, footwear, feet, and baseline vision; the amount of time on the ground or floor; and the inquiry into a recent history of melena were all present in less than 5% of patient charts [10]. Of note, 57% of the patients had complete blood count and electrolyte tests, while only 5% had toxicology screens. Radiographs including X-ray, CT, and MRI were the most common diagnostic tests performed on these patients (79%); 53% were assessed with head CT scans. An attempt at an explanation of the circumstances precipitating the fall was reported for 85% of the patients. The most common cause of falls was environmental factors (43%), followed by aging/functional decline (28%). For 62% of the patients, the fall was described as a "mechanical fall [10]." More than half of all older adult fallers were discharged home. A quarter of patients were admitted to the hospital [10].

Relevance to emergency physicians: This study shows that the ED evaluation of older adult patients with falls was discordant with geriatric fall guidelines. Older patients with more comorbidities may have received more comprehensive evaluations due to several reasons, such as clinicians' biases and the severities of comorbidities, which could lead to a longer length of stay in the ED. The adherence rates for the history and physical examination items recommended by the guidelines were generally poor. Future studies should examine methods to implement the guidelines while minimizing the burden on ED clinicians. While it may not be realistic to have emergency physicians follow every fall recommendation, there is still significant room for improvement. The authors concluded that the current evaluation of older adult fallers in the ED is discordant with general and ED-specific fall guidelines [10].

2. Bhangu J, Hall P, Devaney N, Bennett K, Carroll L, Kenny RA, McMahon CG: The Prevalence of Unexplained Falls and Syncope in Older Adults Presenting to an Irish Urban Emergency Department. Eur J Emerg Med. 2019, 26:100-104 [11]

Summary: This article reveals the probability of an unexplained fall (UEF) and syncope as the cause of falls in elderly patients who present to the ED. UEF is defined as an event where the patient has no recollection of a mechanism to account for the fall. UEF accounted for 20-30% of all ED visits; however, because they are often treated as UEF, syncope has likely been underestimated as a cause. Patients with repeated syncopal episodes were more likely to be treated as cases of UEF. UEF was also said to be associated with greater use of medical resources and hospitalization-related costs. Therefore, the authors analyzed the prevalence, hospitalization patterns, and costs related to UEF. The authors classified patients over 50 years of age who presented with a fall as explained fall (EF), UEF, syncope, or an alternative medical cause. EF is defined as an event that results in a person coming to rest inadvertently on the ground. Alternative medical causes included stroke, witnessed seizure, sepsis, anemia, acute blood loss, and alcohol intoxication. Interestingly, UEF and syncope were less likely to be traumatic. The authors speculate that this may be because syncope is associated with a few seconds of warning beforehand, allowing for self-protective behavior. Of note, 30% of UEF patients had a fall-related ED visit within the past six months; UEF within one year were also five times more likely than EF. Up to 50% of UEF had underlying carotid sinus syndrome, and 20% had an underlying arrhythmia, both considered treatable conditions. Patients with UEF and syncope were twice as likely to be hospitalized and four times more likely to have brain imaging. In this cohort, the cost of hospitalization and diagnostic imaging alone amounted to more than three million Euros; however; the actual cost could be much higher, as the socioeconomic cost for caregivers was not measured. The use of fall and syncope specialists reduced hospitalization rates, length of stay, and unnecessary diagnostic tests, leading to a 36% reduction in subsequent falls. The authors concluded that screening for causes of falls, including syncope, may help prevent a recurrence.

Relevance to emergency physicians: This article highlights UEF and syncope as causes of falls. In particular, when UEF is recognized, syncope should be considered and evaluated aggressively. Finding hidden syncope in the UEF can reduce future falls and hospitalization costs. For this reason, standard syncope assessment and risk stratification for emergency patients are essential to determine the appropriate treatment.

3. Carpenter CR, Avidan MS, Wildes T, Stark S, Fowler SA, Lo AX: Predicting Geriatric Falls Following an Episode of Emergency Department Care: A Systematic Review. Acad Emerg Med. 2014, 21:1069-1082 [12]

Summary: This systematic review and meta-analysis assessed the prognostic accuracy of predictors or constellations of predictors for fall risk in community-dwelling older adults who visit the ED because of a fall [12]. The authors conducted an extensive search for original, prospective, or retrospective ED-based assessments of fall risk in patients who were 65 years and older. Only three out of 608 identified studies met the authors' inclusion criteria. Two studies assessed a total of 29 risk factors and two stratification instruments for falls among 660 older adults in the six months after their ED visits. The third study assessed the risk of falls in the 12 months before an ED visit. The authors found that six characteristics (past falls, living alone, use of walking aid, depression, cognitive deficit, and more than six medications) were assessed as potential risk factors of falls in at least two out of three studies [12]. However, no single risk factor was associated with a statistically significant increase or decrease in fall risk over a six-month period by meta-analysis. A self-report of depression had the highest positive likelihood ratio (LR) for increasing the risk of six-month falls (LR: 6.55, 95% CI: 1.41 to 30.48). In terms of fall risk-screening instruments, Carpenter et al. described a four-item instrument (nonhealing foot sores, self-reported depression, not clipping one's toenails, and previous falls) that yielded optimal predictive accuracy (positive LR of 2.40, 95% CI: 1.95 to 2.8) and a negative LR of 0.11 (95% CI: 0.06 to 0.20) [12].

Relevance to emergency physicians: This systematic review and meta-analysis was selected because it provides a good summary of the findings considered in the fall assessment and their diagnostic accuracy. All ED physicians must be aware that there are no well-validated tools to help us predict future falls. Therefore, it is essential to consider multiple factors when assessing for potential risk factors. Falling is a geriatric syndrome with various iatrogenic, social, physiologic, disease-based, and other factors that can increase the risk. Careful attention to the actual events at the time of the fall may suggest small changes - home safety evaluation, motion detectors for hallway and bathroom lights, discontinuation of bedtime diuretics, grab bars, polypharmacy reduction, and the like - that might improve safety. Future research is needed to better identify fall prediction tools for geriatric patients.

4. Jeanmonod R, Asher S, Roper J, et al.: History and Physical Exam Predictors of Intracranial Injury in the Elderly Fall Patient: A Prospective Multicenter Study. Am J Emerg Med. 2019, 37:1470-1475 [13]

Summary: This paper is a prospective observational study of patients older than 65 years who presented after low-risk falls to three different EDs at two level-one trauma centers with an academic EM program and one community ED. This is a follow-up study of a single-center study that demonstrated historical and physical exam features predicting intracranial injuries (ICI) in geriatric patients with low-risk falls [42]. A low-risk fall was defined as an event where the patients maintained their baseline mental status after the fall and were not triaged to the trauma bay [13]. The features they evaluated were mechanisms of the fall, head trauma history, headache, loss of consciousness (LOC), anticoagulant/antiplatelet use, dementia, and signs of head trauma [13]. A CT scan was obtained at the discretion of treating physicians for the assessment of ICI. Patients were called 30 days post-visit to determine the outcome in non-imaged patients. A total of 723 patients (median age: 83 years, interquartile range: 74-88) were enrolled in this study, but 12 patients were neither imaged nor admitted and were ultimately lost to the follow-up [13]. Of the 711 patients, 21.8% had a history of dementia, and 82.1% had a Glasgow Coma Scale (GCS) score of 15. The traumatic ICI rate was 7.3%; 57% of patients took an anticoagulant or antiplatelet, but this was not a predictor for ICI in this study. Similar to their previous study [42], two variables helped predict ICI: LOC (OR: 2.02, 95% CI: 1.10 to 3.71) and signs of head trauma (OR: 2.6, 95% CI: 1.22 to 5.51). Using these as predictors to screen for ICI, the authors reported 86.5% (95% CI: 73.6 to 94) sensitivity, 38.8% (95% CI: 35.1 to 42.7) specificity, a positive predictive value of 10% (95% CI: 7.5 to 13.3), and a negative predictive value of 97.3% (95% CI: 94.4 to 98.8). Had these items been applied as a decision rule, 273 patients would not have undergone CT, but seven injuries would have been missed. However, none of those injuries required surgical intervention.

Relevance to emergency physicians: Based on these findings, the incidence of ICI identified by CT in older ED patients at their baseline mental status after a fall and not triaged to the trauma bay was relatively low. Signs of trauma to the head and face or LOC were most predictive of ICI. However, most patients did not require neurosurgical intervention. This study reported several risk factors of severe injuries, which could stratify those needing head imaging and potentially observe them without imaging tests. Clinicians could use the prediction model to identify those who do not require head imaging, as sensitivity and negative predictive values are high when stakes are high; for example, when the patient is not interested in head imaging. The magnitude of diagnostic characteristics implies that the prediction model is not accurate enough, so it remains challenging to identify a low-risk group that does not require head imaging. 

5. Goldberg EM, Marks SJ, Resnik LJ, Long S, Mellott H, Merchant RC: Can an Emergency Department-Initiated Intervention Prevent Subsequent Falls and Health Care Use in Older Adults? A Randomized Controlled Trial. Ann Emerg Med. 2020, 76:739-750 [14]

Summary: This randomized controlled trial examined the effect of an ED-initiated intervention to prevent subsequent falls and healthcare use in older adults. ED patients aged 65 years or older who presented to the ED within seven days of a fall were eligible to be included. The intervention included consults with a pharmacist (to change medications that might put the patient at risk of falling) and with a physiotherapist (to assess the risk of falls and develop an action plan for rehabilitation with automated communication to their primary care physicians). The usual care provided patients with a brochure that contained a checklist of home safety measures to prevent falls, but pharmacy and physical therapy consultations were not available. The intervention reduced subsequent fall-related ED visits (adjusted incidence rate ratio: 0.34, 95% CI: 0.15 to 0.76) vs. all ED visits (0.47, 95% CI: 0.29 to 0.74). Of note, this trial did not specify the primary outcome and did not report the number of subsequent falls stated in the trial registry (NCT03360305). Because of the risk of selective reporting, we believe that the trial results are not conclusive but can be hypothesis-generating for future research.

Relevance to emergency physicians: This article is highlighted because it is one of a few interventional studies demonstrating a reduction of falls among older adults seen in the ED. A structured pharmacy and physical therapy review led to a reduction of future falls, which is essential knowledge for any practicing EM provider. However, it is necessary to understand that not all EDs have access to such resources as physiotherapists and pharmacists in the ED. Although this is an important article, it calls for further study to implement a formal medication review and physical therapy evaluation in the ED.

Limitations

Our review has several limitations. Firstly, this review was not intended to be a comprehensive systematic review; instead, it chose to focus on five key articles for those interested in advancing their knowledge of fall care. As such, it is possible that we may have missed some key articles. We sought to reduce this limitation by utilizing social media and expert consultation, as well as a structured literature review. Additionally, this review focused on ED-based fall care, which may have led to the exclusion of topics on fall care in other settings. Finally, it is possible that new literature may have been published since the completion of this review. However, we believe that these articles constitute the landmark papers in the ED-based fall care at the time of this writing.

## Conclusions

We extracted and reviewed five key papers about fall assessment using a modified Delphi process. Appropriate evaluation in the ED for fall patients is crucial. These five articles are of value to EM providers to enhance their care of patients presenting with falls.
